# Risk of venous thromboembolism in patients with rheumatoid arthritis: a meta-analysis of observational studies

**DOI:** 10.1186/s41927-024-00376-9

**Published:** 2024-02-02

**Authors:** Zahra A Fazal, Ana Michelle Avina-Galindo, Shelby Marozoff, Jessie Kwan, Na Lu, J. Antonio Avina-Zubieta

**Affiliations:** 1 Arthritis Research Canada, 230–2238 Yukon Street, BC, V5Y 3P2 Vancouver, Canada; 2 Faculty of Land and Food Systems, University of British Columbia, Vancouver, Canada; 3 Faculty of Science, University of British Columbia, Vancouver, Canada; 4 Division of Rheumatology, Department of Medicine, University of British Columbia, Vancouver, Canada

**Keywords:** Rheumatoid arthritis, Deep vein thrombosis, Venous thromboembolism, Thrombosis, Risk

## Abstract

**Background:**

Thrombotic events, such as venous thromboembolism (VTE) are a major health complication linked to rheumatoid arthritis (RA). We performed a meta-analysis to evaluate the risk of VTE, including deep vein thrombosis (DVT) and pulmonary embolism (PE), in adults with RA compared to the general population.

**Methods:**

MEDLINE and EMBASE databases were searched from inception to April 2022 to identify publications meeting the following criteria: (1) prospective and retrospective original data from cohort or case-control studies; (2) pre-specified RA definition; (3) clearly defined VTE outcomes; (4) reported risk estimate and 95% confidence intervals (95% CIs); (5) at least sex- and age-matched to comparison group; and (6) English language. Of 372 studies screened, 14 were included (602,760 RA patients, 123,076 VTE events) and their quality was assessed by an adaptation of the STROBE quality scoring scale.

**Results:**

The pooled risk ratios of VTE, DVT and PE in patients with RA were 1.57 (95% CI 1.41–1.76), 1.58 (95% CI 1.26–1.97) and 1.57 (95% CI 1.30–1.88), respectively. The I^2^ value of 92%, 94% and 92% for VTE, DVT and PE analyses, suggesting considerable heterogeneity. There were no significant differences in risk estimates among the five subgroup analyses: quality score (*P* = 0.35, I^2^ = 0%); sex (*P* = 0.31, I^2^ = 1.7%); study year (*P* = 0.81, I^2^ = 0%); population source (*P* = 0.35, I^2^ = 0%); study design (*P* = 0.62, I^2^ = 0%).

**Conclusions:**

Results show that patients with RA are at a higher risk of VTE, DVT and PE compared to the general population.

**Supplementary Information:**

The online version contains supplementary material available at 10.1186/s41927-024-00376-9.

## Introduction

Rheumatoid arthritis (RA) is a chronic and disabling autoimmune disease associated with systemic inflammation that affects 1% of the global population [1]. RA causes joint pain, progressive joint destruction and functional limitations, which may affect quality of life of patients [2–4]. Additionally, RA is associated with an increased risk of mortality including from cardiovascular disease among other complications [5]. A major health complication linked to RA is thrombotic events including venous thromboembolism (VTE) [5–8]. VTE, which includes deep vein thrombosis (DVT) and pulmonary embolism (PE), is the third most common vascular event in patients with RA [9].

In RA and other rheumatic diseases, VTE is thought to occur as a complication of inflammation by up-regulating procoagulants, down-regulating anticoagulants, and suppressing fibrinolysis [10–12]. The global burden of thrombosis was estimated to account for 1 to 2 cases of death per 1000 people worldwide in 2016 making it one of the leading causes of death [13–14]. This rate is even higher in patients with chronic inflammatory diseases [25].

Risk estimates for the development of VTE amongst patients with RA in previous studies are wide ranging, from a 1.5 to 6.0-fold increased risk. These risk differences are likely due to methodological limitations and heterogeneity [15–26]. These include population source (clinical vs. community-based), length of follow-up, case and outcome ascertainment, and the use of inception cohorts that can impact the risk estimate of VTE among RA patients [17–19, 27]. To provide a more accurate assessment of this risk by accounting for the factors mentioned above, we conducted a comprehensive systematic review and a meta-analysis of the literature with the objective of evaluating the risk of VTE among patients with RA compared to the general population and assessing sources of heterogeneity.

## Methods

This meta-analysis has been designed according to the 2020 Preferred Reporting Items for Systematic Reviews and Meta-Analyses (PRISMA) statement guideline checklist [28].

### Data sources and searches strategy

A medical librarian systematically searched MEDLINE and EMBASE databases for all eligible studies that identified key published material in English related to the reported risk estimate of DVT, PE, and VTE in adult patients with RA. The original search was conducted from the database inception to October 2020, then updated in April 2022. Records from database searches were downloaded and imported into an EndNote library to facilitate removal of duplicates and screening.

Our search strategy employed mapped subject headings together with keywords for unindexed concepts relating to the themes of RA and VTE. Some major search terms included (but were not limited to): *RA, VTE, DVT, PE, relative risk, and odds ratio (OR).* Search terms were used alone and in combination (e.g., “rheumatoid arthritis” AND “relative risk” or “relative risks” or “odds ratio” or “hazard ratio (HR)” or “incidence rate ratio (IRR)” AND “VTE” or “venous thromboembolism” or “thromboembolic events” or “thrombosis”). The authors also identified additional references via hand-searches of references in relevant papers. A summary of the literature search methodology and resources is provided in Additional File 1.

### Study selection

We reviewed titles and abstracts to select literature that met the following inclusion criteria: *(1)* prospective and retrospective original data from cohort or case-control studies; *(2)* pre-specified RA through methods such as ICD codes, clinical diagnosis (by a general practitioner (GP) or rheumatologist), chart review, American College of Rheumatology classification criteria [29], prescription of antirheumatic drugs; *(3)* clearly defined VTE outcomes through methods such as International Classification of Diseases (ICD) codes, drug prescription (e.g. anticoagulants), clinical diagnosis, chart review, hospital admission data, imaging studies (CT scan, angiography, ultrasound); *(4)* reported risk ratio (RR), odds ratio (OR), hazard ratio (HR), incidence rate ratio (IRR), or standardized incidence ratio (SIR) and corresponding 95% confidence intervals (CI); *(5)* sex- and age-matched or adjusted comparisons from the general population; and *(6)* English language. The exclusion criteria included articles reporting postoperative outcomes, articles that compared the risk of VTE under different rheumatic medications and grey literature including unpublished articles, reports, protocols, editorials, and conference abstracts. Additionally, any previous meta-analyses or meta-syntheses were excluded but their references were searched for relevant studies that met the inclusion criteria. If there were multiple publications using the same study population, the publication with the higher quality, more recent data or with the most detailed information was included.

Two amongst the group of four reviewers (AMA-G, ZAF, SM, JK) were randomly paired and assigned to assess each title and abstract independently through a meta-analysis software, COVIDENCE, and any differences were adjudicated by consensus between the two authors in conflict. Both authors would need to be in consensus that the abstracts met the inclusion criteria to be carried forward for full-text review. The same process was used to independently review all potentially relevant full-text articles against eligibility criteria amongst the same four reviewers and reasons for excluding studies at this stage were recorded. Any conflicts that could not be resolved by each reviewer pair were decided by an independent reviewer (JAA-Z).

### Assessment of quality

To assess the quality of each study, we used an adaptation of the STROBE quality scoring scale for observational epidemiological studies [30]. The quality of each study was assessed by two independent authors (AMA-G, ZAF, SM or JK) using criteria such as the study population, cohort/control selection, comparability and outcome definition with a total of 12 points allowed for cohort studies and 10 points for case-control studies. Any conflicts in the scoring were adjudicated by an independent author (JAA-Z). The quality scoring scale used to evaluate both case-control and cohort studies is included in Additional File 2.

### Data extraction

For each study meeting the eligibility criteria, we extracted following data: author name, year of publication, study reference, country, sample size and population source (e.g., clinical vs. community-based), enrollment period, total number of cases and controls, mean follow-up time, case and outcome ascertainment, total number of VTE (including DVT/PE) events, adjustments for covariates (e.g., age, sex, body mass index, smoking, cholesterol, diabetes mellitus), and the risk estimate with 95% CIs.

### Statistical analysis

This meta-analysis was carried out in Review Manager software (RevMan 5.2), STATA (version 17.0) and HePIMA (version 2.1). The weight-pooled summary of the risk estimates and 95% CIs for VTE outcomes were calculated using the random effects model. The Higgins’s (I^2^) statistic was used to evaluate heterogeneity, whereby 0–40%, 30–60%, 50–90% and 75–100% I^2^ values indicated low, moderate, substantial, and considerable heterogeneity, respectively [31]. Publication bias was assessed with funnel plots [32] and the Egger linear regression test [32]. Subgroup analyses were conducted to evaluate whether the results differed according to the following characteristics: study quality score (≥ 7), sex (male subjects > 25%), year of study (before or after the introduction of JAK inhibitors as a treatment for RA in North America in 2018), population source (community-based vs. clinical-based), and study design (inception vs. non-inception). Subgroup analyses were only conducted for the risk of VTE among patients with RA relative to the general population, meaning that studies that only reported results for the outcomes of DVT and/or PE, but not VTE were not included.

## Results

### Search results

**Fig. 1:**
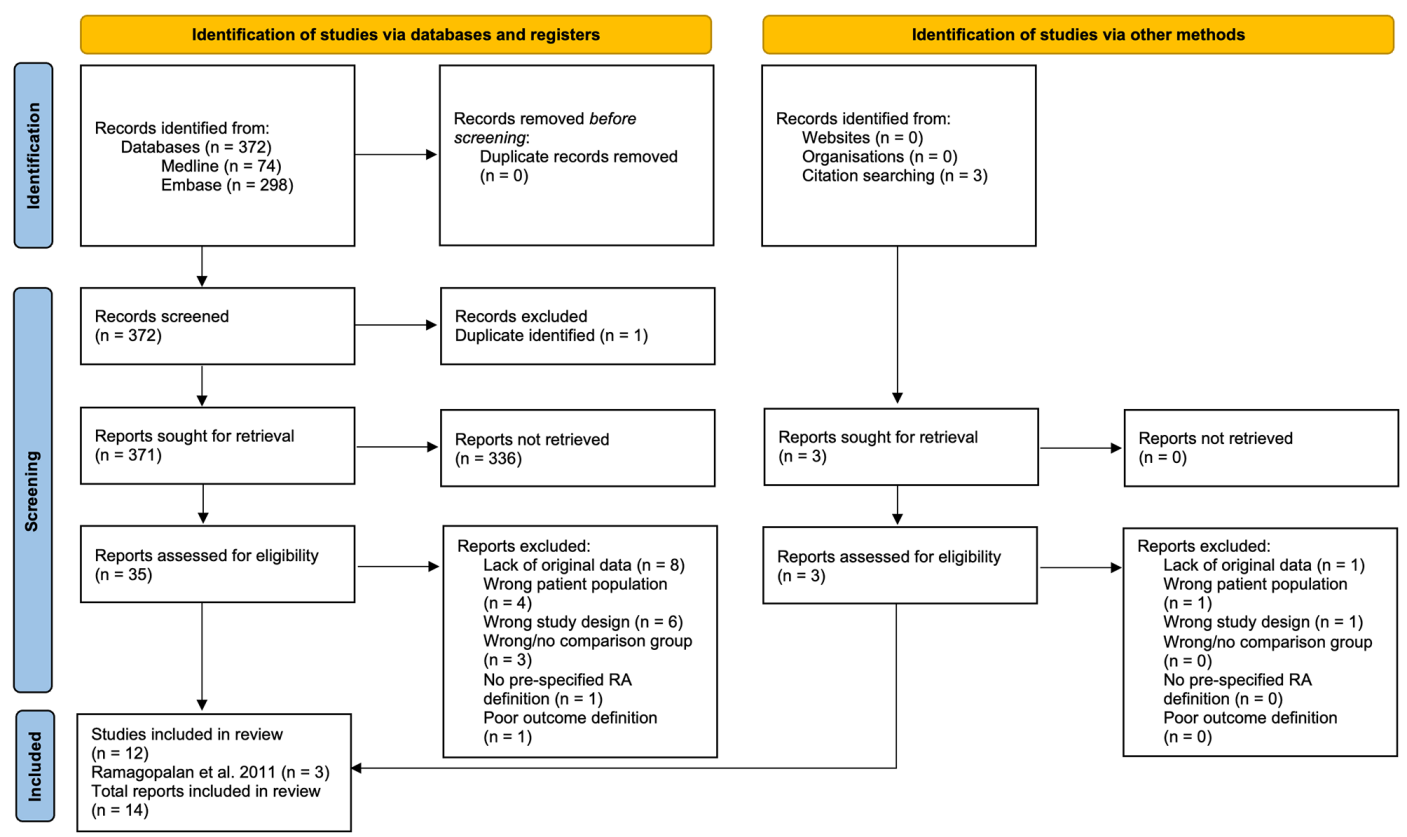
Search methodology and study selection summary

Our first search was conducted in October 2020 and a final search was updated in April 2022, identifying 372 records. After removing duplicates, 371 abstracts published over the last 76 years were identified and selected for title and abstract screening. A total of 35 studies were then retrieved for detailed evaluation of which 12 studies were included in the meta-analysis. The other 23 studies were excluded for the following reasons: eight had no original data, four had the wrong patient population, six had the wrong study design, three had no comparison group, one had no pre-specified RA definition, and one did not have an adequate outcome definition. The paper by Ramagopalan et al. [19] had three cohorts investigating the same RA-VTE association but with different time periods, which we counted individually, increasing the total count of included studies from the original 12 to the final 14. The PRISMA flow diagram (Fig. 1) visually summarizes the study selection process.

#### Study characteristics

The 14 studies included 602,760 RA patients with 123,076 VTE events. 13 were cohort studies and one was a case control study; the characteristics of these studies are provided in Table 1. Nine of these studies were performed in Europe, four in North America and one in Asia. Ten of these studies used community-based samples (*n* = 239,557) and only four were clinical population samples (*n* = 339,793). All studies adjusted for at least age and sex, while eleven of the studies also adjusted for additional potential confounders (e.g., obesity, cancer, bed rest, major surgery, congestive heart failure, varicose veins, fractures, estrogens, stroke, multiple trauma). The mean quality score for the articles included within the cohort studies was 7.67 (range 5–10) on a 12-point scale, and 10 points for the case-control study on a 10-point scale (Table 2).

**Table 1:** Main characteristics of the included studies in the meta-analysis

First author and year	Design	Country	Period	Source of cases	Assessment of cases	Outcome	Assessment of outcomes	Quality score
Bacani 2012	Cohort	USA	1980–2007	EMR	ACR criteria	VTE, PE	MR + lab/imaging	9
Choi 2013	Cohort	UK	1986–2010	EMR	Diagnostic code + DMARD	VTE, DVT, PE	Hospital code + anticoagulant OR hospital code + DC/autopsy	9
Chung 2014	Cohort	Taiwan	1998–2008	Claims data	ACR criteria	DVT, PE	ICD-9 code	10
Holmqvist 2012	Cohort	Sweden	1997–2009	Registry	ACR criteria	VTE, DVT, PE	ICD-10 code	9
Johannesdottir 2012	Case-control	Denmark	1999–2009	Registry	ICD-8/ ICD-10 codes	VTE, DVT, PE	ICD 8–10	9
Kim 2013	Cohort	USA	2001–2008	Claims data	Two physician visits + ICD-9 code + DMARD	VTE, DVT, PE	ICD-9 code + anticoagulants	8
Li 2021	Cohort	Canada	1997–2009	Claims data	Two physician visits + ICD-9 code	VTE, DVT, PE	ICD-9/ ICD-10 + anticoagulants	9
Ramagopalan (a) 2011	Cohort	England, UK	1963–1998	EMR	ICD 7–10 code	VTE	ICD 7–10 code	6
Ramagopalan (b) 2011	Cohort	England, UK	1998–2008	EMR	ICD 7–10 code	VTE	ICD 7–10 code	6
Ramagopalan (c) 2011	Cohort	England, UK	1998–2009	EMR	ICD 7–10 code	VTE	ICD 7–10 code	6
Zoller 2012	Cohort	Sweden	1964–2008	EMR	ICD 7–10 code	PE	ICD 7–10 code	6
Galloway 2020	Cohort	England, UK	1999–2018	EMR	Diagnostic codes	VTE, DVT, PE	Diagnostic codes	8
Molander 2021	Cohort	Sweden	2006–2018	Registry	Diagnosis by clinician	VTE	ICD-10 code	6
Setyawan 2021	Cohort	USA	2014–2018	Claims data	ICD-9 and ICD-10	DVT, PE	Diagnosis by clinician	7

Abbreviations: ACR = American College of Rheumatology; BC = British Columbia; DC = death certificate; DMARDs = disease modifying anti-rheumatic drugs; DVT = deep vein thrombosis; EMR = electronic medical records; ICD = International Classification of Diseases; MR = Medical record; NAV = not available; PE = pulmonary embolism; UK = United Kingdom; USA = United States of America; VTE = venous thromboembolism

**Table 2:** Descriptive statistics regarding venous thromboembolism for included studies

First author and year	Number of RA patients	Number of comparators	Sex (% female)	Mean age or age range	VTE point estimate	DVT point estimate	PE point estimate
Bacani 2012	464	464	69	55.5 ± 15.5	HR 3.6	NAV	NAV
Choi 2013	9589	95,776	69.4	58.3 ± 14.1	RR 2.14	RR 2.16	RR 2.16
Chung 2014	29,238	116,952	77	52.0 ± 15.9	NAV	HR 3.36	HR 2.07
Holmqvist 2012	7904	37,350	69	57.0 ± 15.0	HR 1.6	HR 1.6	HR 1.8
Johannesdottir 2012	1808	147,210	NAV	NAV	IRR 1.2	IRR 1.3	IRR 1.0
Kim 2013	22,143	88,572	75	52.2 ± 12.0	HR 1.4	HR 1.2	HR 1.9
Li 2021	39,142	78,078	65.8	59.5 ± 15.7	HR 1.28	HR 1.3	HR 1.25
Ramagopalan (a) 2011	14,231	313,716	73	NAV	RR 1.45	NAV	NAV
Ramagopalan (b) 2011	11,241	187,609	72	NAV	RR 1.57	NAV	NAV
Ramagopalan (c) 2011	268,005	3,707,315	71	NAV	RR 1.75	NAV	NAV
Zoller 2012	82,020	NAV	70.5	NAV	NAV	NAV	SIR 1.91
Galloway 2020	23,410	93,640	71.1	59	HR 1.54	HR 1.64	HR 1.57
Molander 2021	46,316	215,843	74	NAV	RR 1.88	NAV	NAV
Setyawan 2021	47,249	182,431	NAV	NAV	NAV	IRR 1.04	IRR 1.08

Abbreviations: DVT = deep vein thrombosis; HR = hazard ratio; IRR = incidence rate ratio; PE = pulmonary embolism; NAV = not available; RA = rheumatoid arthritis; RR = risk ratio; VTE = venous thromboembolism

### Overall risk of venous thromboembolism, deep vein thrombosis, and pulmonary embolism

**Fig. 2:**
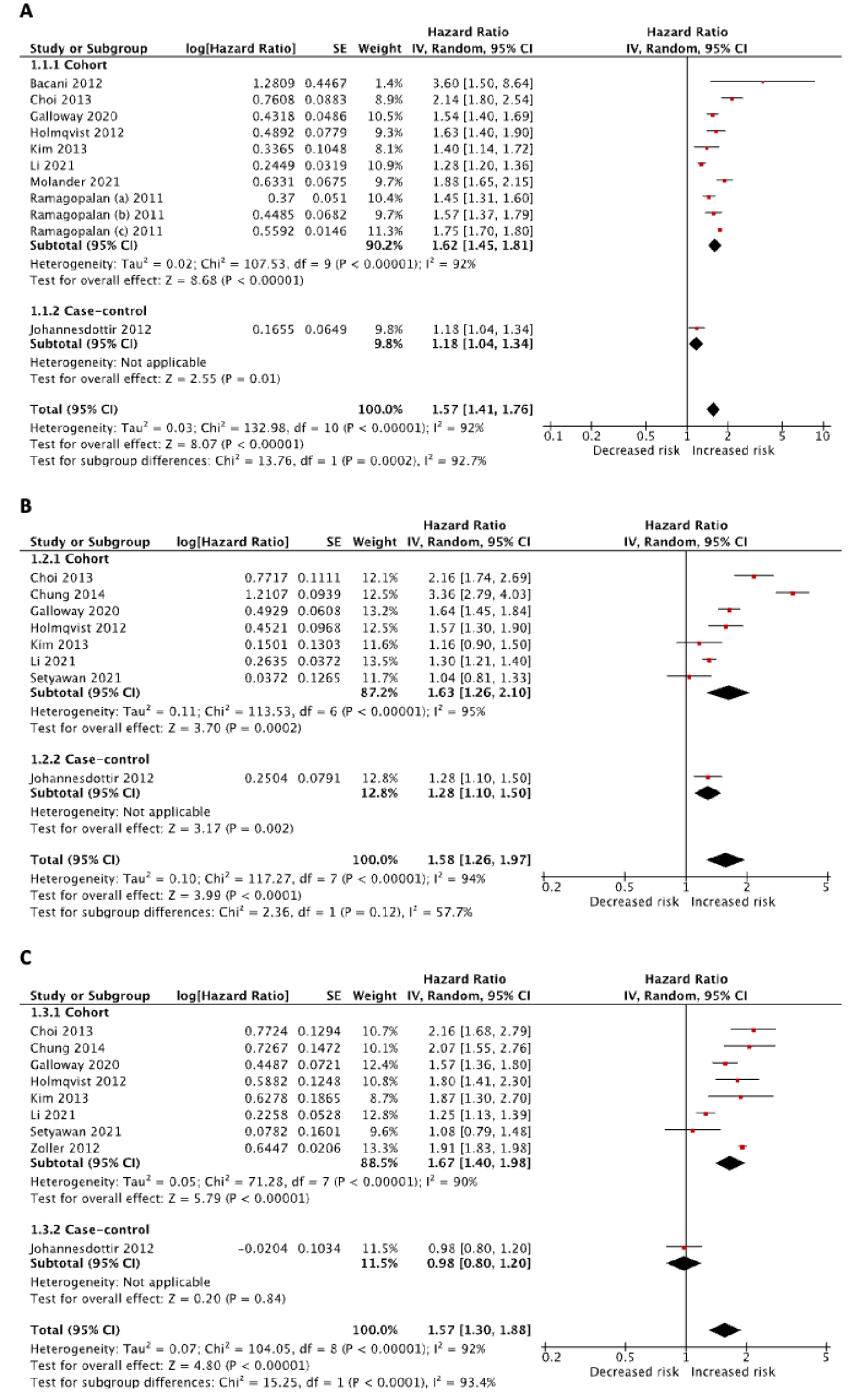
Forest plot for (**A**) venous thromboembolism, (**B**) deep vein thrombosis, (**C**) pulmonary embolism outcomes in patients with rheumatoid arthritis compared to the general population

In total, 11 studies (*n* = 444,253 RA cases) assessed the overall risk of VTE, eight studies (*n* = 180,483 RA cases) assessed the overall risk of DVT, and nine studies (*n* = 262,503 RA cases) assessed the overall risk of PE (Fig. 2A-C). The risk of incident VTE was significantly increased by 57% in patients with RA compared to the general population (pooled RR 1.57 (95% CI 1.41–1.76)). There was a 58% increase in the risk of DVT in patients with RA (pooled RR 1.58 (95% CI 1.26–1.97)). There was a 57% increase in the risk of PE in patients with RA (pooled RR 1.57 (95% CI 1.30–1.88)). The I^2^ value was 92%, 94%, and 92% for VTE, DVT, and PE analyses, suggesting considerable heterogeneity (Fig. 2A-C). Results stratified by study design are shown in Fig. 2A-C. The test for subgroup differences comparing cohort and case-control studies was statistically significant for the VTE (*p* = 0.0002) and PE (*p* = 0.0001) outcomes, but not for DVT (*p* = 0.12). Additional results regarding heterogeneity for VTE are presented in Additional File 3.

### Publication bias

**Fig. 3:**
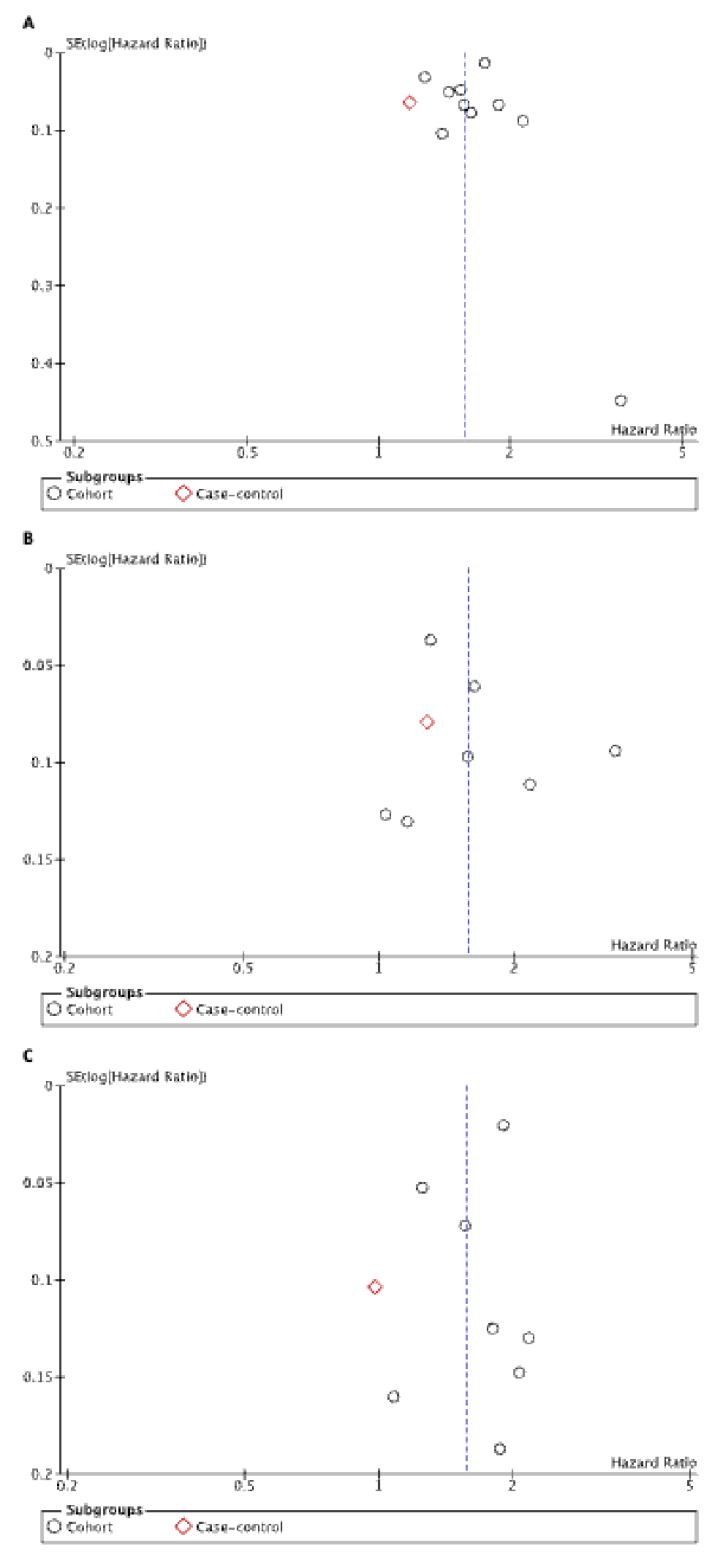
Funnel plot evaluating the risk of (**A**) venous thromboembolism, (**B**) deep vein thrombosis, (**C**) pulmonary embolism outcomes in patients with rheumatoid arthritis compared to the general population. Each dot represents an individual study. The horizontal line is the random-effects pooled estimate of log (HR).

The funnel plots for VTE and PE showed some asymmetry indicating publication bias in small studies (Fig. 3A-C). The VTE funnel plot showed one study occupying the southeastern part of the plot while the PE funnel plot showed more studies occupying the right-hand side of the overall effect line. The P value of Egger’s test for both these outcomes was > 0.05, indicating that publication bias was negligible in our meta-analysis. The funnel plot for DVT had an approximate symmetry to the distribution and this indicated no presence of publication bias, although the horizontal spread of studies is wide.

### Subgroup analyses

To investigate potential causes of heterogeneity, subgroup analyses were conducted stratified by quality score for the individual studies (≥ 7), sex (% male participants > 25%), year of study (before or after the introduction of JAK inhibitors as a treatment for RA in North America in 2018), population source (community-based vs. clinical-based), and study design (inception vs. non-inception). Of the 14 studies included in our meta-analysis, 11 reported results for VTE and were included in subgroup analyses. There were no significant differences among any of the five subgroup analyses: quality score (*P* = 0.35, I^2^ = 0%); sex (*P* = 0.31, I^2^ = 1.7%); study year (*P* = 0.81, I^2^ = 0%); population source (*P* = 0.35, I^2^ = 0%); study design (*P* = 0.62, I^2^ = 0%) (Additional File 4). Within all subgroup analyses, pooled risk estimates indicated a statistically significant increased risk of VTE among patients with RA relative to the general population. Additionally, heterogeneity, as measured by the I^2^ statistic, remained high (> 75%) in all subgroups.

## Discussion

From this meta-analysis, the pooled risk estimates of VTE, DVT, and PE in patients with RA were found to be ~ 60% for all outcomes indicating that patients with RA had a higher risk of VTE compared to the general population. This increased risk remained significant even when stratified by quality score for the individual studies (≥ 7), sex (% male participants > 25), year of study, population source (community-based vs. clinical-based), and study design (inception vs. non-inception), meaning that these variables did not modify the effect of RA on VTE.

Our meta-analysis builds on a previously published meta-analysis by Ungprasert et al. in 2014 on the risk of VTE in patients with RA [33]. As reported in their paper, the authors identified nine relevant publications and reported pooled RRs of 1.96 (95% CI 1.81–2.11), 2.17 (95% CI 2.05–2.31), and 2.08 (95% CI 1.75–2.47) for VTE, PE, and DVT, respectively, for patients with RA versus patients without RA. Similarly, another meta-analysis from 2014 by Lee & Pope assessed the risk of VTE in inflammatory rheumatic diseases [34]. Their findings indicated a positive association between RA and VTE, with a resultant OR of 2.23 (95% CI 2.02–2.47) from 10 included studies, comparing patients with RA to age-, sex-, and other comorbidity-matched populations. More recently, Hu et al. conducted a meta-analysis in RA patients relative to healthy controls, finding pooled ORs of 2.23 (95% CI 1.79–2.77), 2.15 (95% CI 1.39–3.49), and 2.25 (95% CI 1.70–2.98) for VTE, PE, and DVT, respectively [35]. We have aimed to expand on this prior work by utilizing more stringent entry criteria (e.g., excluding older publications that used the same dataset as a more recent publication, excluding cross-sectional studies). Additionally, we planned subgroup analyses, given that previous meta-analyses had identified moderate or high between-study heterogeneity in their analyses. However, our five subgroup analyses were unable to explain the considerable heterogeneity.

The etiology of VTE events in RA is still unclear, although the mechanisms are likely multifactorial. Virchow’s triad of vascular injury, hypercoagulation, and venous statis has been used to describe the basic mechanisms leading to thrombosis. It is believed that systemic inflammation induced by RA may affect at least two of these three components: vascular injury and hypercoagulation. Regarding vascular injury, a proinflammatory state in the early disease stages has been demonstrated to lead to activation of endothelial cells, altered endothelial permeability, and increased leukocyte and platelet adhesion [36]. In terms of hypercoagulability, inflammation modulates a thrombotic response by upregulating procoagulants, downregulating anticoagulants, and suppressing fibrinolysis [12]. There is insufficient evidence at present assessing the role of venous statis and VTE risk in patients with RA. However, a number of acute-phase reactants, such as C-reactive protein, erythrocyte sedimentation rate, fibrinogen, coagulation factor VIII, and von Willebran factor, are elevated in inflammatory disease patients and also known to increase plasma viscosity, a risk factor for thrombosis [37].

Among patients with RA, the risk of VTE is greatest in the first year after diagnosis, and then decreases thereafter [16, 18, 21]. The evidence suggests that earlier stages of RA involve higher levels of inflammation, after which the maximum benefits of antirheumatic therapy are achieved. However, even with newer and more potent antirheumatic therapies, recent studies demonstrate that patients with RA have an increased risk of VTE years after diagnosis. For instance, Li et al. found that within five years after diagnosis, patients with RA were still at an increased risk compared to individuals without RA (HR 1.28 (95% 1.20–1.36)) [21].

Anti-inflammatory therapies may affect the relationship between RA and VTE. Over the past several decades, advances in the treatment and management of RA have occurred, including the introduction of inflammation-reducing medications such as anti-TNF- α agents beginning in the 1990 and 2000 s in North America, and the shift towards a treat-to-target approach in 2010, which targets remission or low disease activity [38, 39]. These introductions may impact the relationship between RA and VTE, although we were unable to assess this, given that studies included in our meta-analysis had long follow-up times, covering the introduction of these treatments. Quasi-experimental study designs such as interrupted time series would be well-suited to studying the effect of the introduction of anti-inflammatory medications and the treat-to-target approach on VTE outcomes.

Other VTE risk factors are also present among individuals with RA. Smoking and obesity are commonly observed in RA patients [38]; similarly, major surgery (e.g., knee, hip) and hospitalization with immobilization are more common, and risk factors for VTE [40]. Certain RA therapies are also known to increase the risk of VTE, including cyclooxygenase-2 inhibitors (2-fold increase in risk) [41] and glucocorticoids (2-3-fold increase in risk for short-term use) [42]. The evidence is still mixed for certain other RA medications, including JAK inhibitors. In a pooled analysis of four phase III placebo-controlled trials of baricitinib in patients with RA, an imbalance was reported in VTE incidence for 4 mg daily dose versus 2 mg daily dose or placebo (1.3 and 0 events per 100 patient-years, respectively) [43]. However, a recent meta-analysis by Yates et al. found that VTE risk was not increased among patients with immune-mediated inflammatory diseases taking JAK inhibitors relative to placebo groups [44].

Our study findings have practical implications. Given the increased risk of VTE, including DVT and PE, among patients with RA, strategies for the primary prevention of VTE are suggested. Patients with RA may require additional monitoring by health care providers, particularly during high-risk periods such as immediately after diagnosis or after surgery. Increased awareness of this risk may need to be communicated to patients with RA with other VTE risk factors, as listed above and VTE prophylaxis could be discussed between clinicians and patients. Finally, additional research is needed for biomarkers related to this increased risk, as well as early VTE identification strategies and improved treatments.

The strengths of our meta-analysis are the rigorous methods used to conduct the review, extensive literature search by an experienced medical librarian, and a reproducible search strategy, extraction, and synthesis. We had a stringent approach to study inclusion, wherein the majority of the studies included were high-quality. Our findings provide an accurate and contemporary assessment of the risk of VTE among individuals with RA by accounting for the factors mentioned above. Our results can have implications for the clinical management of patients with RA, potentially improving morbidity and mortality rates.

There are limitations that should be mentioned. The degree of heterogeneity among all analyses and subgroup analyses is a shortcoming. Some degree of heterogeneity was expected due to factors such as the difference in assessment of VTE outcome, risk factors with VTE and their adjustment within studies as well as the extent to which studies were consistent in their definition of cases and outcomes. Some of the heterogeneity in our results can be attributed to the number of cohort studies included within our meta-analysis. The large number of cohort studies included may have skewed our data but these have the benefit of avoiding recall bias and reducing the possibility of selection bias or reverse causation. We were unable to perform multiple meta-regression to examine this heterogeneity due to the small number of included studies. The funnel plots for VTE and DVT outcomes also showed asymmetry indicating possible publication bias. In subgroup analyses, no statistically significant difference of effect was demonstrated for any of the subgroups and the pooled effect remained significantly elevated in each subgroup. Thus, this meta-analysis provides a more comprehensive investigation on the risk of VTE in patients with RA compared to the general population by accounting for factors that have traditionally resulted in a wide range of risk estimates. Future research should build on these findings to investigate the role of inflammation in the association between RA and VTE.

## Conclusions

Our meta-analysis demonstrates that there is a statistically significant increased risk of VTE among patients with RA compared to the general population. As VTE is associated with a high morbidity and mortality amongst patients with RA, our study suggests that physicians should carefully monitor patients with RA for VTE.

### Electronic supplementary material

Below is the link to the electronic supplementary material.

Supplementary Material 1

## Data Availability

All data generated or analyzed during this study are included in this published article and its additional files.

## Abbreviations

95% CI: 95% confidence interval
DVT: Deep vein thrombosis
GP: General practitioner
HR: Hazard ratio
ICD: International Classification of Diseases
IRR: Incidence rate ratio
OR: Odds ratio
PE: Pulmonary embolism
PRISMA: Preferred Reporting Items for Systematic Reviews and Meta-Analyses
RA: Rheumatoid arthritis
RR: Risk ratio
SIR: Standardized incidence ratio
VTE: Venous thromboembolism
