# Supplementary material for: Risk of venous thromboembolism in patients with rheumatoid arthritis: a meta-analysis of observational studies
Source: BMC Rheumatol. 2024 Feb 2;8:5. doi: 10.1186/s41927-024-00376-9 (PMC10836002; doi:10.1186/s41927-024-00376-9)
Supplement: Supplementary file 1 — Supplementary Material 1 [file 41927_2024_376_MOESM1_ESM.pdf]

Additional File 1: PRISMA 2020 Checklist

Section and Topic	Item #	Checklist item	Location where item is reported
**TITLE**			
Title	1	Identify the report as a systematic review.	1
**ABSTRACT**			
Abstract	2	See the PRISMA 2020 for Abstracts checklist.	2-3
**INTRODUCTION**			
Rationale	3	Describe the rationale for the review in the context of existing knowledge.	4
Objectives	4	Provide an explicit statement of the objective(s) or question(s) the review addresses.	4-5
**METHODS**			
Eligibility criteria	5	Specify the inclusion and exclusion criteria for the review and how studies were grouped for the syntheses.	6
Information sources	6	Specify all databases, registers, websites, organisations, reference lists and other sources searched or consulted to identify studies. Specify the date when each source was last searched or consulted.	5
Search strategy	7	Present the full search strategies for all databases, registers and websites, including any filters and limits used.	Supplementary material
Selection process	8	Specify the methods used to decide whether a study met the inclusion criteria of the review, including how many reviewers screened each record and each report retrieved, whether they worked independently, and if applicable, details of automation tools used in the process.	6-7
Data collection process	9	Specify the methods used to collect data from reports, including how many reviewers collected data from each report, whether they worked independently, any processes for obtaining or confirming data from study investigators, and if applicable, details of automation tools used in the process.	7
Data items	10a	List and define all outcomes for which data were sought. Specify whether all results that were compatible with each outcome domain in each study were sought (e.g. for all measures, time points, analyses), and if not, the methods used to decide which results to collect.	6
	10b	List and define all other variables for which data were sought (e.g. participant and intervention characteristics, funding sources). Describe any assumptions made about any missing or unclear information.	6
Study risk of bias assessment	11	Specify the methods used to assess risk of bias in the included studies, including details of the tool(s) used, how many reviewers assessed each study and whether they worked independently, and if applicable, details of automation tools used in the process.	7
Effect measures	12	Specify for each outcome the effect measure(s) (e.g. risk ratio, mean difference) used in the synthesis or presentation of results.	6
Synthesis methods	13a	Describe the processes used to decide which studies were eligible for each synthesis (e.g. tabulating the study intervention characteristics and comparing against the planned groups for each synthesis (item #5)).	6-7
	13b	Describe any methods required to prepare the data for presentation or synthesis, such as handling of missing summary statistics, or data conversions.	8
	13c	Describe any methods used to tabulate or visually display results of individual studies and syntheses.	8
	13d	Describe any methods used to synthesize results and provide a rationale for the choice(s). If meta-analysis was performed, describe the model(s), method(s) to identify the presence and extent of statistical heterogeneity, and software package(s) used.	8
	13e	Describe any methods used to explore possible causes of heterogeneity among study results (e.g. subgroup analysis, meta-regression).	8
	13f	Describe any sensitivity analyses conducted to assess robustness of the synthesized results.	8
Reporting bias assessment	14	Describe any methods used to assess risk of bias due to missing results in a synthesis (arising from reporting biases).	8
Certainty assessment	15	Describe any methods used to assess certainty (or confidence) in the body of evidence for an outcome.	None

Additional File 1: PRISMA 2020 Checklist

Section and Topic	Item #	Checklist item	Location where item is reported
**RESULTS**			
Study selection	16a	Describe the results of the search and selection process, from the number of records identified in the search to the number of studies included in the review, ideally using a flow diagram.	8-9
	16b	Cite studies that might appear to meet the inclusion criteria, but which were excluded, and explain why they were excluded.	8-9
Study characteristics	17	Cite each included study and present its characteristics.	9 and Table 1
Risk of bias in studies	18	Present assessments of risk of bias for each included study.	9 and Table 1
Results of individual studies	19	For all outcomes, present, for each study: (a) summary statistics for each group (where appropriate) and (b) an effect estimate and its precision (e.g. confidence/credible interval), ideally using structured tables or plots.	Table 2
Results of syntheses	20a	For each synthesis, briefly summarise the characteristics and risk of bias among contributing studies.	14-15 and Table 1
	20b	Present results of all statistical syntheses conducted. If meta-analysis was done, present for each the summary estimate and its precision (e.g. confidence/credible interval) and measures of statistical heterogeneity. If comparing groups, describe the direction of the effect.	14-15
	20c	Present results of all investigations of possible causes of heterogeneity among study results.	15
	20d	Present results of all sensitivity analyses conducted to assess the robustness of the synthesized results.	15
Reporting biases	21	Present assessments of risk of bias due to missing results (arising from reporting biases) for each synthesis assessed.	14
Certainty of evidence	22	Present assessments of certainty (or confidence) in the body of evidence for each outcome assessed.	None
**DISCUSSION**			
Discussion	23a	Provide a general interpretation of the results in the context of other evidence.	15-6-16
	23b	Discuss any limitations of the evidence included in the review.	19
	23c	Discuss any limitations of the review processes used.	19
	23d	Discuss implications of the results for practice, policy, and future research.	18
**OTHER INFORMATION**			
Registration and protocol	24a	Provide registration information for the review, including register name and registration number, or state that the review was not registered.	None
	24b	Indicate where the review protocol can be accessed, or state that a protocol was not prepared.	None
	24c	Describe and explain any amendments to information provided at registration or in the protocol.	None
Support	25	Describe sources of financial or non-financial support for the review, and the role of the funders or sponsors in the review.	1
Competing interests	26	Declare any competing interests of review authors.	1
Availability of data, code and other materials	27	Report which of the following are publicly available and where they can be found: template data collection forms; data extracted from included studies; data used for all analyses; analytic code; any other materials used in the review.	None

From: Page MJ, McKenzie JE, Bossuyt PM, Boutron I, Hoffmann TC, Mulrow CD, et al. The PRISMA 2020 statement: an updated guideline for reporting systematic reviews. BMJ 2021;372:n71. doi: 10.1136/bmj.n71

For more information, visit: http://www.prisma-statement.org/

Additional File 2: Systematic search methodology

Research Question(s)

What is the risk of deep venous thrombosis, pulmonary embolism and venous thromboembolism in adult patients with rheumatoid arthritis?

Items Identified by Database

Database Name	Number of items Identified	Number of items (Duplicates Removed)
Medline	77	74
Embase	339	298
Total	416	372

Example of Search

Embase 1974 to 2022 Week 13, Ovid MEDLINE(R) ALL 1946 to April 01, 2022

1	exp Venous Thrombosis/	202376
2	exp deep vein thrombosis/	130839
3	(deep vein thrombos* or deep-vein thrombos* or deep venous thrombos* or deep-venous thrombos* or deep phlebothrombos* or deep thrombophlebitis).ti,ab,kf,kw.	78469
4	DVT.ti,ab,kf,kw.	35270

5	or/1-4 (DEEP VEIN THROMBOSIS (DVT))	229980
6	exp Pulmonary Embolism/	147956
7	lung embolism/	106114
8	(pulmonary embolis* or pulmonary emboliz* or pulmonary thromboembolis* or pulmonary thromboemboliz* or pulmonary embolus or pulmonary microembolus or pulmonary microembolis* or pulmonary microemboliz* or pulmonary thromboembolic disease or lung embolis* or lung emboliz* or lung thromboembolis* or lung thromboemboliz* or lung embolus or lung microembolus or lung microembolis* or lung microemboliz* or lung thromboembolic disease).ti,ab,kf,kw.	115355
9	or/6-8 (PULMONARY EMBOLISM (PE))	178149
10	Venous Thromboembolism/	58550
11	venous thromboembolism/	58550
12	(venous thromboembolism* or vein thromboembolism* or VTE).ti,ab,kf,kw.	73018
13	or/10-12 (VENOUS THROMBOEMBOLISM (VTE))	90165
14	exp "Arthritis, Rheumatoid"/	340773
15	exp rheumatoid arthritis/	340773
16	(rheumatoid arthritis or inflammatory arthritis or rheumatic arthritis or Caplan Syndrome or "Caplan's Syndrome" or Caplans Syndrome	338480

	or Felty Syndrome or "Felty's Syndrome" or Rheumatoid Nodul* or Rheumatoid Vasculitis or Rheumatoid Vasculitides or "Sjogren's Syndrome" or Sjogrens Syndrome or Sjogren Syndrome or Sicca Syndrome or "Adult-Onset Still's Disease" or Adult-Onset Still Disease).ti,ab,kf,kw.	
17	or/14-16 (RHEUMATOID ARTHRITIS (RA))	434625
18	5 and 17 (RA + DVT)	2049
19	9 and 17 (RA + PE)	1419
20	13 and 17 (RA + VTE)	812
21	or/18-20 (RA + CONDITIONS)	3276
22	exp risk/	4135671
23	relative risk.ti,ab,kf,kw.	162893
24	or/22-23 (RELATIVE RISK)	4204191
25	21 and 24 (RA + CONDITIONS + REL RISK)	958
26	Odds Ratio/	119470
27	(odds ratio or risk ratio or odds ratios or risk ratios or relative odds or cross-product ratio or cross-product ratios).ti,ab,kf,kw.	797499
28	or/26-27 (ODDS RATIO)	839801
29	21 and 28 (RA + CONDITIONS + ODDS RATIO)	177
30	25 or 29 (RA + CONDITIONS + REL RISK/ODDS RATIO)	1015

31	Cohort Studies/	999428
32	cohort analysis/	1135399
33	Longitudinal Studies/ or Follow-Up Studies/ or Prospective Studies/ or Retrospective Studies/	4834050
34	Case-Control Studies/	468076
35	case control study/	505997
36	(cohort stud* or cohort analys* or longitudinal stud* or follow up stud* or prospective stud* or retrospective stud* or case control stud*).ti,ab,kf,kw.	2184296
37	or/31-36 (OBSERV STUDIES)	6412968
	Embase <1974 to 2022 April 01>	3592572
	Ovid MEDLINE(R) ALL <1946 to April 01, 2022>	2820396
38	30 and 37 (RA + CONDITIONS + REL RISK/ODDS RATIO + OBSERV STUDIES)	428
39	(case study or case studies).ti,ab,kf,kw.	259924
40	30 and 39	0
41	(editorial or comment or letter or newspaper article).pt.	4005916
42	38 not 41	416
	Embase <1974 to 2022 April 01>	339
	Ovid MEDLINE(R) ALL <1946 to April 01, 2022>	77
43	remove duplicates from 42	361

	Embase <1974 to 2022 April 01>	284
	Ovid MEDLINE(R) ALL <1946 to April 01, 2022>	77
44	from 43 keep 285-361 (MEDLINE)	77
45	from 43 keep 1-284 (EMBASE)	284
46	(conference or conference abstract or conference paper or conference review or congresses).pt.	5141665
47	45 not 46 (EMBASE NO CONFERENCES)	171
48	45 not 47 (EMBASE ONLY CONFERENCES)	113

Additional File 3: Quality scoring scale for cohort and case-control studies

QUALITY SCORING FOR COHORT STUDIES

**Cohort:**	**2**	**1**	**0**
Study Population	Community	Clinical	Not described
Cohort sample	Inception cohort	Not inception cohort	Not described
RA definition	ACR	Other, but validated	Defined, but not validated
Diagnosis of VTE outcome	Validated criteria	Non validated criteria	Not mentioned
RA exposure	> 10 years	> 5yrs, <10 years	<10 years
Loss to follow-up	less than 20%	20-40%	>40% or not explained
Matching or adjustment for	> 6 VTE risk factors (*)	< 6 VTE risk factors	None or unexplained

(*) age, sex, body mass index, smoking, cholesterol, diabetes mellitus
Community: is a non-selected sample (random sample, population-based (databases that include all cases)
Clinical: usually patients seen in one or several clinics, hospitals (selected samples that do not represent everyone)
Inception cohort: all cases seen at the disease onset or within the same time period (i.e. within the first year of disease) and then follow them (usually incident cases)
ACR: America College of Rheumatology criteria
RA exposure: disease duration (follow-up time)
(*) risk factors for VTE: age > 40 yrs, obesity, cancer, bed rest, major surgery, congestive heart failure, varicose veins, fractures, estrogens, stroke, multiple trauma (MVA), childbirth, MI.

QUALITY SCORING FOR CASE CONTROL STUDIES

**Case-control:**	**2**	**1**	**0**
Definition of VTE cases	Validated criteria	Other, but not validated	
Definition of RA	ACR criteria for RA	Other, but validated	Defined, but not validated
Controls selection	Same population as cases	Different from cases	Not clear
Incident cases of VTE	Yes	Do not know	
Response rate	>80% of cases and contr.	60-80% of cases and contr.	<60% or not explained
Matching or adjustment for	4 out of 5 Framingham (*) risk factors	less than 4 Framingham (*) risk factors	None or unexplained

(*) age, sex, hypertension, smoking, total cholesterol, diabetes mellitus

Additional File 4: Heterogeneity of the results

Table 1. Statistics under the fixed effects model and the random effects model for venous thromboembolism outcome, n=11 studies

**Model**	**Pooled risk ratio**	**95% confidence interval**	**P-value**
Fixed effects model	1.63	1.59, 1.67	<0.00001
Random effects model	1.57	1.41, 1.76	<0.00001

Table 2. Heterogeneity statistics for venous thromboembolism outcome, n=11 studies

**Statistic**	**Value**
$\tau 2$	0.03
Ri*	0.96
CVB**	0.37

*Ri is the proportion of total variance due to between-studies variance

**CVB is the coefficient of variation between studies

Table 3. Asymptomatic heterogeneity tests for venous thromboembolism outcome, n=11 studies

**Statistic**	**Value**	**Degrees of fredom**	**P-value**
Q; χ^2	166.76	10	<0.0001
Z2wls; F *	1228.64	(1,10)	<0.0001
Z2wls,r; χ^2 *	3.67	1	0.0555
Z2k; F *	2.70	(1,10)	0.1317

Table 4. Bootstrap tests for venous thromboembolism outcome, n=11 studies

**Statistic**	**P-value**
Q	<0.0001
Z2wls	<0.0001
Z2wls,r	0.1720
Z2k	0.0050
*T*2 (alpha=0.05)	Significant

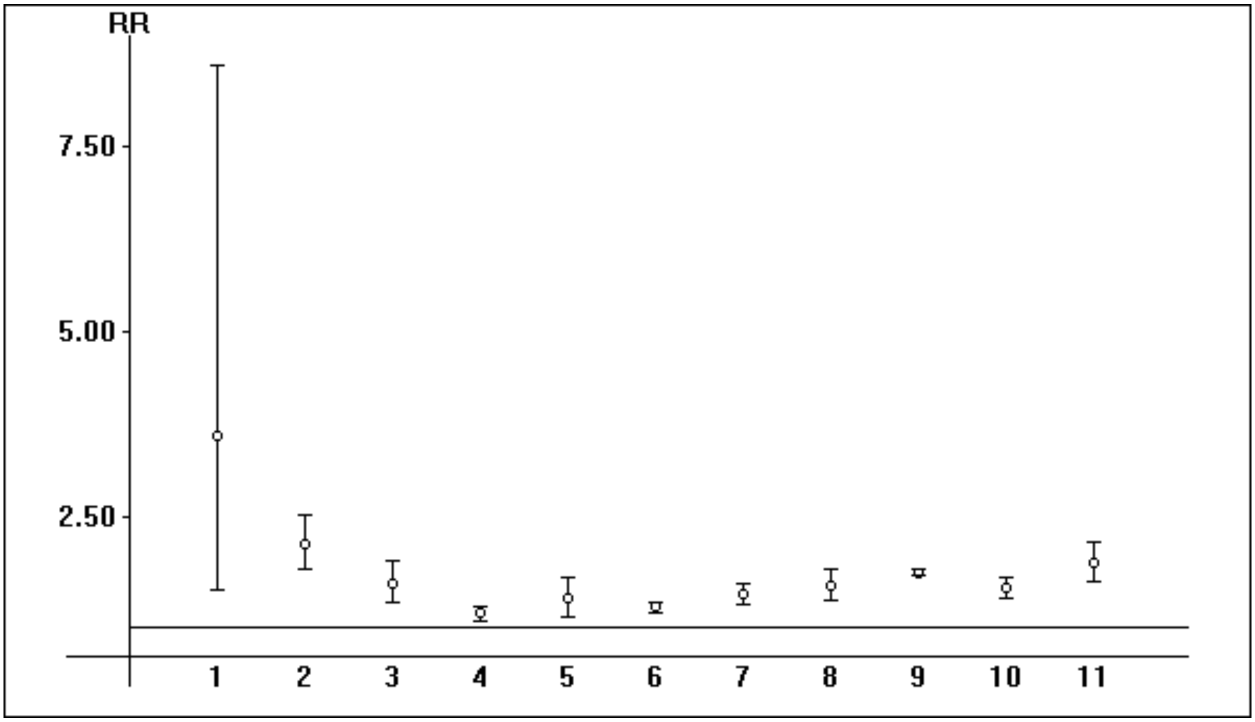

Figure 1. Odd man out graph for venous thromboembolism outcome, n=11 studies

Additional File 5: Subgroup analyses

Subgroups	# of studies	Overall effect		Heterogeneity		Subgroup differences	
		Effect estimate (95% CI)	P-value	X^2	I^2 (%)	P-value	I^2 (%)
	11	1.57 (1.41, 1.76)	<0.05	132.98	92%		
Quality score						0.35	0%
High quality	7	1.52 (1.41, 1.75)	<0.05	49.68	88%		
Low quality	4	1.66 (1.49, 1.84)	<0.05	16.44	82%		
Sex						0.31	1.7%
<25% Male	1	1.40 (1.13, 1.73)	<0.05	N/A	N/A		
≥25% Male	10	1.59 (1.41, 1.78)	<0.05	133.89	93%		
Study year						0.81	0%
2018 or after	3	1.54 (1.24, 1.75)	<0.05	31.25	94%		
2017 or earlier	8	1.58 (1.40, 1.80)	<0.05	60.97	89%		
Population source						0.35	0%
Community	7	1.52 (1.31, 1.76)	<0.05	49.68	88%		
Non-community	4	1.66 (1.49, 1.84)	<0.05	16.44	82%		

Study design						0.62	0%
Inception	4	1.74 (1.29, 2.35)	<0.05	38.68	92%		
Non-inception	6	1.61 (1.47, 1.75)	<0.05	24.71	80%		

95% CI: 95% confidence interval
VTE: venous thromboembolism
Johannesdottir et al. (2012) was excluded from the study design from the subgroup analysis, as it is a case-control study
